# Development of High-Protein Vegetable Creams by Using Single-Cell Ingredients from Some Microalgae Species

**DOI:** 10.3390/foods10112550

**Published:** 2021-10-22

**Authors:** Fatma Boukid, Josep Comaposada, Albert Ribas-Agustí, Massimo Castellari

**Affiliations:** 1 Food Safety and Functionality Programme, Institute of Agriculture and Food Research and Technology (IRTA), 17121 Monells, Spain; albert.ribas@irta.cat (A.R.-A.); massimo.castellari@irta.cat (M.C.); 2 Food Quality and Technology Programme, Institute of Agriculture and Food Research and Technology (IRTA), 17121 Monells, Spain; josep.comaposada@irta.cat

**Keywords:** viscosity, color, nutritional labeling, spirulina, Chlorella, Tetraselmis, Nannochloropsis, nutrition claims, vegan

## Abstract

The aim of this paper was to develop high-protein vegetable creams through the incorporation of microalgae. Single-cell ingredients from *Arthrospira* *platensis* (spirulina), *Chlorella vulgaris*, *Tetraselmis chui*, and *Nannochloropsis oceanica* were incorporated at two levels of addition (1.5% and 3.0%) to a standard vegetable cream (STD). Effects of incorporation were assessed in terms of physicochemical and rheological attributes as well as nutritional labeling facts. Creams formulated with 3% *A. platensis*, *N. oceanica*, or *T. chui* showed strong color differences (6 < ΔE < 12) compared to STD; creams formulated with 1.5% *A. platensis*, *T. chui*, or *N. oceanica* showed perceptible differences (3 < ΔE < 6); and those made with *C. vulgaris* at 1.5 and 3% exhibited small differences (ΔE < 2). Moisture content, water activity, pH, syneresis, and °Brix did not show significant changes. Adding microalgae increased Bostwick consistency and decreased the consistency coefficient (K) except in creams made with *A. platensis*, which showed comparable values to STD. Principal component analysis indicated that creams made with 1.5% *C. vulgaris* were the most similar to STD considering all evaluated parameters. Estimation of the nutritional labeling facts showed that the four formulations could be labeled as having “high protein content” following the present EU legislation.

## 1. Introduction

Vegetables creams are traditional, flavorful, and nutritious foods usually served as a side dish or a snack. The global market of vegetable creams is rapidly growing and projected to reach USD 21.0 billion by 2027 because of several drivers [1]. Changing preferences about the consumption of healthy food products are boosting the development of innovative vegetable cream products. Clean-label and natural trends are urging manufacturers to use natural ingredients, avoiding all type of additives (e.g., hydrocolloids, emulsifiers, preservatives, colorants, and thickeners) [2]. The coronavirus 2019 outbreak contributed to changing eating habits, since consumers were more aware about the relatedness between health and nutrition, resulting in more consumption of vegetable-based foods. Vegan and vegetarian lifestyles greatly contributed into this market expansion for ethical reasons (animal welfare and sustainability concerns). Flexitarian consumers, having growing concerns over meat consumption and its association with health problems, also have a relevant impact in shifting the consumption of plant-based products, including creams [3].

Currently, there is a wide variety of cream products in the market with different forms such canned, ready-to-eat, and powder [4]. Canned products are condensed and require adding water and cooking prior to consumption. Dry soup mixes are reconstituted with hot water before eating, and some fresh ingredients and spices can be added to improve the flavor. Dried soup powders have the advantage of flavor stability for a long period (up to 1 year) [5]. Ready-to-eat creams are cheap and convenient and require microwaving for a few minutes prior to eating. The latter category is witnessing great growth, since it responds to modern consumer lifestyles by offering convenience and ease of preparation. However, in some cases, ready-to-eat creams might contain high amounts of starchy ingredients, saturated fats, or additives. The new trend in ready-to-eat vegetable cream products is the development of nutritious and health-promoting products. Nutritional profile can be improved by introducing protein, mineral, and vitamin sources from plant origin that are suitable for a wide target of consumers or by reducing the amount of fat, sugar, and salt [6,7,8]. Functional and nutrient content claims are of increasing relevance for consumers making the purchase decision [9]. High protein is among the top claims driving the growth of several food and beverage products [10]. Vegetable proteins are versatile and derive from several sources such as cereals, vegetables, pulses, and algae (seaweed and microalgae). A claim that a food is high in protein may only be made where at least 20% of the energy value of the food is provided by protein [11].

Microalgae are seen as a forerunner resource to close the so-called “protein gap”. Sought in both freshwater and marine environments, microalgae find extensive utilization as food as well as live feed in aquaculture [12,13]. Gaining popularity as a promising nutrient source, microalgae impart high nutrition and are used as a high-protein supplement in human nutrition [14,15,16,17,18]. *Arthrospira* *platensis* (commercially known as Spirulina) (Cyanobacteria) or *Chlorella vulgaris* (Chlorophyta) are the most used species as food ingredients, mainly because of their long history of use, while companies using other species must apply to the Novel Food Regulation (EU) 2015/2283 before commercialization [19]. However, saline microalgae species, such as *Tetraselmis chui* (Chlorophyta) (already approved for food) or *Nannochloropsis oceanica* (Ochrophyta, Eustigmatophyceae) (approved only for use as feed in the EU), grown in marine water (and therefore with a more sustainable water footprint), might replace freshwater species in the mid-/long-term. *N. oceanica* is a source of protein but is mostly known for its rich composition in long-chain fatty acids such as eicosapentaenoic acid, which have several health benefits [20,21]. *T. chui* contains 46.5 g/100 g dry weight (DW) protein with an essential amino acid index of 0.9 [22], reflecting its enormous potential as a dietary source of sustainable protein. However, its application in foods is still limited because of several challenges such as palatability, sensory acceptance, and regulatory approval as a general food ingredient [23]. 

Microalgae such as *A. platensis* or *C. vulgaris* have been used in different products such as bread [24], yogurt [25,26], biscuits [27], ice creams [28], soups [29], puddings/gelled desserts [30], and pasta [31]. Nevertheless, to the best of the authors’ knowledge, no studies have focused on the use of these microalgae in reformulating vegetable creams. In the present study, the aim was to study the effects of the incorporation of single-cell ingredients from four microalgae species on the physicochemical characteristics of vegetable creams. From this perspective, the reformulation design considered two factors: microalgae species (*A. platensis, C. vulgaris, T. chui*, and *N. oceanica*) and two levels of addition (1.5 and 3%). The quality of the final products was evaluated in terms of physicochemical properties (color, water activity, moisture content, pH, brix, and syneresis), rheological properties, and nutritional labeling compared to a standard formulation (STD, no microalgae addition).

## 2. Materials and Methods

### 2.1. Vegetable Cream Preparation

Frozen vegetables (spinach, zucchini, chickpea, leek, broccoli, and chard) were bought from Geland (Girona, Spain). Single-cell microalgae ingredients (dried powder form of microalgae produced in closed photobioreactors) were provided by Necton (Olhão, Portugal) and Allmicroalgae (Pataias, Portugal), and the rest of ingredients (mineral water, sunflower oil, and salt) were purchased from a local supermarket. The chemical composition of the microalgae ingredients, as indicated in their label, is reported in Table S1. The vegetables used are standard ingredients commonly used in commercial vegetable cream preparation.

Vegetable creams were prepared according to nine different formulations, including the standard recipe (STD, without microalgae ingredients) and two recipes for each microalgae species with two levels of inclusion (1.5 and 3.0%, calculated on the basis of the total weight of the vegetable ingredients, keeping constant the amounts of water, oil, and salt) (Table 1). The level of substitution of microalgae was computed to be retrieved proportionally from each vegetable.

All ingredients were weighed and cooked at 90 °C for 25 min with continuous mixing at 300 rpm by a cooking robot (Thermomix^®^, Vorwerk, Wuppertal, Germany). Afterwards, the product was homogenized for 75 s by progressively increasing the mixing speed from 2000 to 7600 rpm. The creams were packaged in 200 mL glass bottles, left to cool down until reaching 20 °C, and then heat-treated in an autoclave (Ilpra Systems, Mataró, Spain) at 116 °C/75 min/1.2 Bar.

Three independent replicates (1 kg) were produced for each formulation, giving a total of 27 cream samples.

### 2.2. Vegetable Cream Characterization

#### 2.2.1. Physicochemical Analysis

Samples (30 g) were spread on glass petri dishes (BRAND^®^ Petri dish, Sigma-Aldrich, Madrid, Spain). Photos of the creams were taken in a darkroom system using a digital camera (Canon 50D E05, Canon, Tokyo, Japan) associated to a computer using SpectroMagic software (Konica Minolta, Inc., Osaka, Japan) using the following settings: 90 mm focal length, ISO 100, F11, and M 1/15.

Color parameters L* (Lightness), a* (degree of redness), and b* (degree of yellowness) were measured with a CR-410 D65 colorimeter (Minolta Co., Osaka, Japan). Total color difference (ΔE) was calculated using Equation (1):

(1) $$\Delta E=\sqrt{{\left(L_{sample}^{*}-L_{STD}^{*}\right)}^{2}+{\left(a_{sample}^{*}-a_{STD}^{*}\right)}^{2}+{\left(b_{sample}^{*}-b_{STD}^{*}\right)}^{2}}$$

where:

L*_sample_, a*_sample_, b*_sample_ = color parameters of vegetable cream;

L*_STD_, a*_STD_, b*_STD_ = color parameters of standard vegetable cream.

Moisture content (MC, % g water/100 g product) was determined by weight loss by drying the samples (10 g) in a forced-air oven (J.P. Selecta, Abrera, Spain) at 102 °C to a constant weight. Crude protein content (N 6.25) was determined by the Kjeldahl method after acid digestion. Water activity of the vegetable creams was measured at 25 °C (Aqualab 4TE, Decagon Devices Inc., Pullman, WA, USA). The pH was measured at 25 °C (HACH Company, Loveland, CO, USA). Syneresis was performed by centrifuging 30× *g* of cream at 14,500 rpm/15 min at 4 °C. After this phase, the supernatant was collected and weighed. The analysis was done in triplicate for each cream category. The result was expressed as a percentage (g supernatant per 100 g cream) [32]. °Brix of the supernatant was measured at 20 °C with a handheld refractometer (Atago, ATC 1E, Tokyo, Japan).

All analyses were carried out in duplicate for each production, resulting in 6 determinations for each formulation.

#### 2.2.2. Rheological Analysis

Bostwick consistency was measured at 20 °C before sterilization with a Bostwick consistometer (ZXCON-CON3, PCE Instruments Ibérica S.L., Albacete, Spain). The Bostwick consistometer sample chamber was filled with 100 mL of product, and then the gate of the chamber was released to allow vegetable cream to flow. The distance (cm) travelled by the sample was recorded after 30 s [33].

The vegetable creams’ apparent viscosity was measured with a rotational rheometer (Haake 550 Rheostress 1, Thermo Electric Corporation, Karlsruhe, Germany) with a type Z34DIN Ti sensor system of coaxial cylinders. Shear stress (σ) was recorded according to the shear rate (γ) from 0 to 800 s^−1^. The analysis was performed with the sterilized vegetable cream at 50 °C to mimic product serving temperature. The data were fitted to the Oswald de Waele model using RheoWin Data Manager (RheoWin Pro V. 4.0, HAAKE, Karlsruhe, Germany), and consistency coefficient (K) and flow behavior index (*n*) values were calculated according to Equation (2).

(2) $$\sigma =K\gamma^{n}$$

#### 2.2.3. Nutritional Labeling

Nutritional label of creams was computed based on data provided on commercial product labels or technical sheets (Table S1). Energy value was calculated using the energy factors provided in EU Regulation nº 1169/2011 [34], in which carbohydrates (excluding polyols), protein, fats, and fibers account for 4 kcal/g, 4 kcal/g, 9 kcal/g, and 2 kcal/g, respectively.

### 2.3. Statistical Analysis

Multivariate analysis of variance (MANOVA) was used to determine the effect of microalgae species (S) and level of addition (LA) on the physicochemical properties and rheological properties of vegetable creams. MANOVA was performed based on fixed factors using Pillai’s trace test. The percentages of total variations were computed to determine the contribution of the factors (S and LA) and their interactions in the variance of each parameter. The percentage of total variation was computed to explain the variance of each parameter as a function of the sum squares of the main factors and their interaction. Significant differences among the mean values were calculated using Duncan’s test. A principal component analysis (PCA) was performed based on the correlation matrix. All experimental data were statistically analyzed using SPSS version 19.0 (SPSS Inc., Chicago, IL, USA).

## 3. Results and Discussion

### 3.1. Physicochemical Evaluation

The effect of microalgae species and its level of addition on the color of vegetable cream products is summarized in Table 2. Microalgae species (S) significantly influenced all the parameters considered, while the level of addition (LA) affected all parameters except water activity. Significant interaction between the two independent factors was also evidenced for the color parameters, pH, and syneresis. L* was controlled mainly by S (∼56%), followed by LA (∼38%) and S × LA (∼7%); a* was chiefly controlled by S (∼98%), with small but significant impacts of LA (∼1%) and S × LA (∼0.61%); and b* was controlled mainly by S (∼71%), followed by LA (∼24%) and S × LA (∼6%).

**Figure 1:**
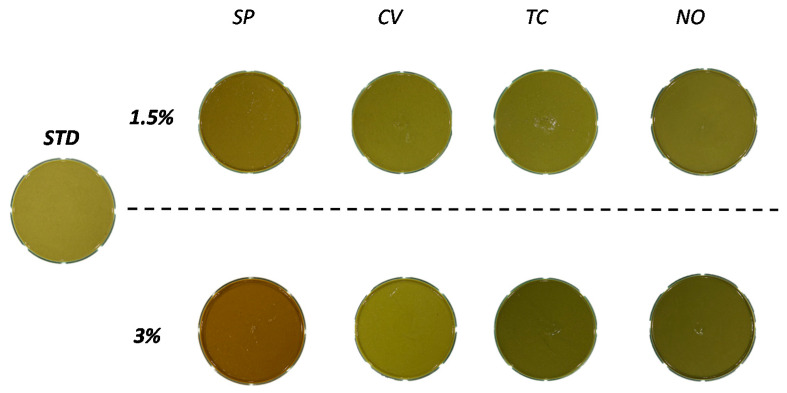
Photos of vegetable creams reformulated with different microalgae at different levels of additions. STD: standard, SP: Spirulina (*Arthrospira platensis*), CV: *Chlorella vulgaris*; TC: *Tetraselmis chui*; NO: *Nannochloropsis oceanica*.

As illustrated in Table 3, the addition of microalgae significantly decreased L* values, resulting in microalgae-containing creams with darker color compared to STD (Figure 1). These results are consistent with the impact of microalgae on the color of broccoli soups [29]. Creams made with *C. vulgaris* were the most similar to STD because of the lighter color of *C. vulgaris* powder compared to the rest of the microalgae powders.

Increasing the level of addition induced a significant decrease in L* regardless of species. The parameter a* was negative for all the studied samples, indicating a greenish color, except for *A. platensis*, which had positive a* values (and thus a reddish color). Compared to STD, creams enriched with *C. vulgaris* were the most similar in a*, while those made with *A. platensis* were the most different, as illustrated in Figure 1. This can be attributed to the high pigmentation of *A. platensis* powder [35]. The impact of addition level did not have a pattern (an increase or decrease), but it significantly affected the change of a* values for each species. Regarding yellow index (b*), formulations enriched with *C. vulgaris* (1.5 and 3%) had the highest values, even higher than STD. Regarding LA effect, creams made with 3% microalgae had lower b* than those made with 1.5% for all species.

ΔE was used to determine whether the total color difference of a sample from STD was visually observable. Table 2 revealed that ΔE was controlled by S (∼56%), LA (∼35%), and S × LA (∼10%). Based on Table 3, creams formulated with *A. platensis* 3%, *N. oceanica* 3%, and *T. chui* 3% showed strong color differences (6 < ΔE < 12) compared to STD. This suggests that the color differences between these microalgae-containing creams and STD were visible to the human eye [29]. Creams formulated with *A. platensis*, *T. chui*, and *N. oceanica* with a level of addition of 1.5% showed perceptible differences (3 < ΔE < 6) compared to STD. On the other hand, creams made with *C. vulgaris* at 1.5% and 3% exhibited only small differences in cream color (ΔE < 2).

Moisture content (MC) was significantly controlled by LA (∼90%), while the effect of S (∼9%) was less pronounced and no significant effect was found for the interaction. Increasing LA induced a slight reduction in MC of creams made with all species. This can be attributed to the substitution of frozen vegetables with high moisture content by a dry microalgae powder. Water activity (a_w_) showed small differences among the studied formulations (0.988–0.992, Table 3). These changes resulted mainly from S (∼69%; Table 2), and no pattern was observed by increasing the level of addition. This can be related to salt content differences between freshwater (*A. platensis* and *C. vulgaris*) and saline (*T. chui* and *N. oceanica*) species. Indeed, LA and S × LA did not significantly affect this parameter. The values of pH ranged from 5.86 to 6.16 (Table 3). Although the changes in pH were minimal, they were controlled mainly by S (∼92%). In fact, *A. platensis* and *C. vulgaris* had the lowest value, since they were freshwater species and were therefore the most similar to STD, while *N. oceanica* and *T. chui* were saline water species with higher pH values. LA and S × LA had less impact (3% and 5%, respectively) on pH. Indeed, increasing LA increased the pH of creams made with *T. chui* and *N. oceanica*, while no effect was found in creams made with *C. vulgaris* and *A. platensis*.

Syneresis was significantly controlled by S (∼46%), LA (∼32%), and S × LA (∼22%) (Table 2). Regarding the effect of S, all creams had syneresis values lower than that of STD. *C. vulgaris* 3% was the most similar to STD. Increasing LA induced a significant decrease in syneresis for all species except *T. chui* (no significant effect of LA) (Table 3).

°Brix was controlled mostly by LA (∼83%), followed by S (∼12%), while no significant interaction effect was found. Indeed, increasing LA increased brix because of the increase in the total soluble solids due to more microalgae in the formulation. Creams made with 1.5% microalgae content were statistically similar to STD regardless of the species (Table 3).

### 3.2. Rheological Properties

**Figure 2:**
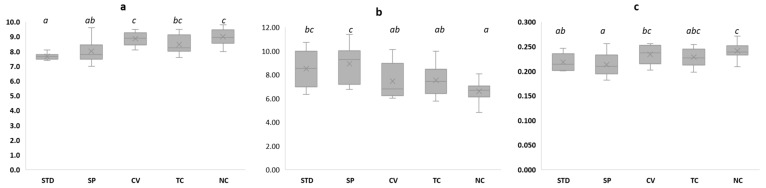
Rheological properties of vegetable creams as a function of microalgae. STD: standard; SP: Spirulina (*Arthrospira platensis*); CV: *Chlorella vulgaris*; TC: *Tetraselmis chui*; NO: *Nannochloropsis oceanica*. (**a**) Bostwick consistency (cm); (**b**) consistency coefficient (K); (**c**) flow behavior index (n). Different letters (a–c) in different bars indicate significant (*p* < 0.05) differences between samples for the given parameter.

As illustrated in Table 2, rheological properties were impacted only by microalgae species (S), and no significant effect of level of addition (LA) or S × LA was found. The influence of S on the Bostwick consistency, consistency coefficient (K), and flow index (n) was high (~80%, ~86%, and ~89%, respectively; Table 2). Similar behavior was observed in other semisolid products, such as ice creams, enriched with microalgae [28]. This can be attributed to the differences in physicochemical composition of microalgae species (dry matter, fat, and protein content, Table S1) affecting the rheological characteristics of the resulting creams. To underline the impact of species, rheological parameters are presented as a function of S in Figure 2.

Bostwick consistency slightly increased after the addition of microalgae, indicating a decreased consistency as compared to STD, but remained within the industrial range (6–10 cm). Microalgae addition probably induced changes in matrix component interactions, resulting in a reduction in network strength. Similar behavior due to changes in the combination between proteins and polysaccharides was observed in batter enriched with microalgae [36].

Consistency coefficient (K) values ranged between 6.44 and 9.49. Compared to Bostwick consistency (measured before sterilization at 20 °C), it was expected that K values would decrease because of increasing temperature (measured after sterilization at 50 °C) [37]. However, K did not show a constant pattern compared to Bostwick consistency, probably because of the different behavior of microalgae species with different thermophysical properties [38]. *A. platensis* had the highest K, followed by STD, *C. vulgaris, T. chui*, and *N. oceanica* (Figure 2). Higher K values indicate a more pronounced viscous characteristic, which corresponds to cream with stronger network structure [39]. This result can be likely attributed to the functional properties of *A. platensis* contributing into the formation of thicker creams compared to the other species [30]. Indeed, proteins isolated from *A. platensis* have potential use in formulating foods with higher viscosities or gels, in which said proteins could act as fillers to strengthen networks [40]. Similarly, in the case of batter and yogurt made with *A. platensis*, viscosity increased because of the high water-holding capacity of the polysaccharides, fibers, and protein in the microalgae, making the product more viscous [36,41,42]. The presence of phycocyanin protein molecules may have contributed to increased viscosity in *A. platensis*-based creams [43]. Sulphated polysaccharides of *A. platensis* were also found to act as a gelling agent and stabilizer [44]. On the other hand, *N. oceanica*, having the highest lipid content, showed the lowest K because of the negative impact of lipids on the emulsification behavior of proteins/emulsifiers. This is consistent with the reduced K of ice cream products prepared with fat-rich microalgae such as *Diacronema* *vlkianum (Haptophyta, Pavlovophyceae)* [28]. Therefore, microalgae addition might affect the consistency of creams and improve quality as a multifunctional ingredient because of the biochemical composition of microalgae [28].

The flow behavior index (n) was lower than 1 (and ranged between 0.18 and 0.28) in all samples, indicating a pseudoplastic (shear thinning) behavior (Figure 2). *N. oceanica* had the highest value, followed by *C. vulgaris, T. chui*, STD, and *A. platensis*. The vegetable creams made with *C. vulgaris, T. chui*, STD, and *A. platensis* showing lower shear thinning behavior could result in more stability against forces applied during pumping and mixing of the products [28]. Therefore, the use of *C. vulgaris, T. chui,* and *A. platensis* can be stated to produce a more desirable texture compared to the use of *N. oceanica*. Cream properties can also be modulated by the addition of protein, gums, or starches with different functionalities to maintain the original viscoelastic properties of vegetable creams [39].

### 3.3. Principal Component Analysis

**Figure 3:**
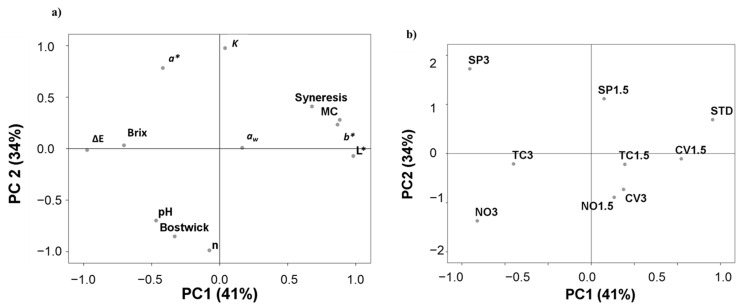
Scattering the data of physicochemical and rheological properties by the first two principal components (PC1 and PC2) derived from principal component analysis (PCA) of vegetable creams reformulated using different strains at different levels of addition. (**a**) Biplot of the first two components; (**b**) rotated principal scores of reformulated vegetable creams projected into the first two principal components. STD: standard; SP: Spirulina (*Arthrospira platensis*); CV: *Chlorella vulgaris*; TC: *Tetraselmis chui*; NO: *Nannochloropsis oceanica*.

A principal component analysis was performed to visualize the effects of S and LA and to identify the most similar formulation to STD (Figure 3). The total accumulative variance from the first two principal components accounted for 75% of the total variance; the first component accounted for 41%, and the second component accounted for 34%. The first component was expressed as function of L*, ΔE, MC, b*, brix, syneresis, and aw, while the second component was expressed as a function of n, K, Bostwick consistency, a*, and pH (Figure 3a). The projection of the cream formulations on the factorial space is illustrated in Figure 3b. No clear discrimination was observed as a function of species. As a function of LA, all formulations made with 1.5% microalgae were located on the same side as STD (the positive side of PC1), while those made with 3% were located on the opposite side except for creams made with 3% *C. vulgaris*. Overall, cream reformulated with 1.5% *C. vulgaris* was most similar to STD.

### 3.4. Nutritional Labeling

Estimation of the nutritional labeling facts of the nine creams is reported in Table 4. Increasing microalgae content resulted in an increase in energy likely due to the additional fat and carbohydrate contributions. Furthermore, saturated fatty acids and sugars were slightly increased compared to STD, while unsaturated fatty acids remained almost stable. Protein content (calculated using the database or determined using the Kjeldahl method) increased in all the samples with added microalgae compared to STD. All the formulations could be labeled as “source of proteins” (in which protein provides more than 12% of the energy value), while four creams (i.e., 1.5% and 3% *A. platensis*, 3% *T. chui*, and 3% *N. oceanica*) could be labeled as “high protein” products (in which protein provides more than 20% Kcal). All products could be labeled as “high fiber” (in which fiber is higher than 3 g/100g Kcal). These results suggest microalgae as a functional ingredient to produce high-protein creams. Besides the approved species, *N. oceanica* can be considered a potential alternative for developing protein-rich foods if approved for food consumption.

## 4. Conclusions

Based on the obtained results, the incorporation of microalgae did not hinder the physicochemical or rheological properties of vegetable creams. Nevertheless, adding 1.5% and 3% of four different microalgae species induced significant changes compared to STD. Color was significantly changed; creams made with *C. vulgaris* were the most similar to STD, while those made with 3% *N. oceanica, T. chui*, and *A. platensis* presented observable changes. Species and level of addition had significant effects on moisture content, water activity, pH, syneresis, and brix, but the obtained creams had values within the same range of STD. Rheological properties were impacted mainly by microalgae species. Increasing microalgae content reduced creams’ consistency except in creams made with *A. platensis*. Overall, creams made with 1.5% *C. vulgaris* were the most similar to STD based on PCA results. Nutritional labeling showed that four formulations (1.5% and 3% *A. platensis*, 3% *N. oceanica*, and 3% *T. chui*) could be claimed as high-protein products. These results would boost the use of microalgae as ingredients in vegetable creams as a rich source of protein. Further studies will be performed to explore the organoleptic properties of selected formulations and go deeper investigating the health-beneficial effects of microalgae-containing creams.

## Figures and Tables

**Table 1:** Formulation of the vegetable creams containing microalgae (g/100 g).

Code	Microalgae	Microalgae	Vegetables	Water	Oil	Salt
STD	Standard recipe	0	67.6	29	2.9	0.5
SP1.5	*A. platensis* 1.5% *(w/w)*	1.01	66.59			
CV1.5	*C. vulgaris* 1.5% *(w/w)*					
TC1.5	*T. chui* 1.5% *(w/w)*					
NO1.5	*N. oceanica* 1.5% *(w/w)*					
SP3	*A. platensis* 3% *(w/w)*	2.03	65.87			
CV3	*C. vulgaris* 3% *(w/w)*					
TC3	*T. chui* 3% *(w/w)*					
NO3	*N. oceanica* 3% *(w/w)*					

**Table 2:** Multivariate analysis of the quality characteristics of vegetable creams reformulated with different microalgae and two levels of addition.

	Microalgae Species (S)		Level of Addition (LA)		S × LA	
	SS%	Significance	SS%	Significance	SS%	Significance
Physicochemical properties						
L*	55.53	***	37.64	***	6.82	***
a*	98.11	***	1.28	***	0.61	***
b*	70.66	***	23.72	***	5.62	***
ΔE	56.11	***	33.84	***	10.05	***
Moisture content	8.95	***	90.31	***	0.74	ns
a_w_	69.16	**	1.00	ns	29.84	ns
pH	91.96	***	2.71	***	5.33	***
Syneresis	45.95	***	32.12	***	21.93	***
°Brix	11.96	*	82.67	***	5.37	ns
Rheological properties						
Bostwick consistency (cm)	80.05	***	15.80	ns	4.15	ns
Consistency coefficient (K)	85.56	**	0.03	ns	14.41	ns
Flow behavior index (*n*)	89.00	*	2.35	ns	8.65	ns

ns: not significant; *: *p* ≤ 0.05; **: *p* ≤ 0.01; ***: *p* ≤ 0.001; SS: sum of squares.

**Table 3:** Physicochemical properties of vegetable creams reformulated with different microalgae and two levels of addition.

	L*	a*	b*	ΔE	Moisture Content (%, g H20/100 g Sample)	*a_w_*
STD	44.84 ± 0.80 g	−2.05 ± 0.12 d	22.96 ± 0.13 g	0.00 ± 0.00 a	87.51 ± 0.30 e	0.990 ± 0.001 b
SP1.5	40.44 ± 0.12 c	0.57 ± 0.07 f	21.91 ± 0.42 c	5.24 ± 0.06 d	86.66 ± 0.16 cd	0.990 ± 0.002 ab
SP3	37.15 ± 0.32 a	1.17 ± 0.14 g	18.66 ± 0.33 bc	9.39 ± 0.13 f	85.58 ± 0.39 ab	0.991 ± 0.001 b
CV1.5	43.74 ± 0.99 f	−2.19 ± 0.08 c	23.83 ± 0.90 h	1.74 ± 0.74 b	86.77 ± 0.16 d	0.991 ± 0.001 b
CV3	42.95 ± 0.37 e	−2.20 ± 0.05 e	23.43 ± 0.43 gh	1.99 ± 0.45 b	85.77 ± 0.04 b	0.991 ± 0.001 b
TC1.5	41.64 ± 0.34 d	−2.63 ± 0.13 a	20.72 ± 0.34 e	3.97 ± 0.29 c	86.68 ± 0.13 cd	0.990 ± 0.002 ab
TC3	38.11 ± 0.20 b	−2.32 ± 0.08 b	17.37 ± 0.37 b	8.76 ± 0.16 e	85.82 ± 0.21 b	0.988 ± 0.001 a
NO1.5	41.50 ± 0.28 d	−2.20 ± 0.03 c	19.32 ± 0.48 d	4.96 ± 0.38 d	86.42 ± 0.24 c	0.992 ± 0.002 b
NO3	37.57 ± 0.71 ab	−1.86 ± 0.11 e	16.20 ± 0.44 a	9.96 ± 0.33 g	85.33 ± 0.37 a	0.990 ± 0.001 b
	**pH**	**Syneresis** **(g Supernatant** **per 100 g Cream)**	**Brix**	**Bostwick** **Consistency** **(cm)**	**Consistency Coefficient (K, Pa s^n^)**	**Flow Behavior Index (n)**
STD	5.88 ± 0.03 a	66.29 ± 1.75 d	4.43 ± 0.393 a	7.67 ± 0.24 a	8.52 ± 1.66 bc	0.218 ± 0.019 ab
SP1.5	5.87 ± 0.02 a	64.63 ± 0.90 c	4.37 ± 0.225 a	7.92 ± 0.41 ab	8.36 ± 1.78 bc	0.217 ± 0.021 ab
SP3	5.86 ± 0.03 a	62.49 ± 0.43 b	5.15 ± 0.084 d	8.10 ± 1.08 abc	9.49 ± 1.35 bc	0.210 ± 0.027 a
CV1.5	5.86 ± 0.03 a	65.44 ± 1.06 cd	4.57 ± 0.234 a	8.65 ± 0.50 bcde	7.74 ± 1.57 ab	0.229 ± 0.018 abc
CV3	5.86 ± 0.02 a	62.39 ± 1.18 b	4.97 ± 0.052 cd	9.08 ± 0.33 de	7.16 ± 1.36 ab	0.239 ± 0.022 bc
TC1.5	6.01 ± 0.01 b	64.39 ± 0.93 c	4.58 ± 0.293 ab	8.38 ± 0.81 abcd	7.70 ± 1.61 ab	0.227 ± 0.022 abc
TC3	6.05 ± 0.02 c	65.11 ± 0.19 c	5.22 ± 0.117 d	8.55 ± 0.43 bcde	7.34 ± 0.93 ab	0.230 ± 0.015 abc
NO1.5	6.04 ± 0.03 c	63.16 ± 0.55 b	4.35 ± 0.176 a	8.70 ± 0.49 cde	6.77 ± 1.17 ab	0.238 ± 0.022 bc
NO3	6.16 ± 0.04 d	60.40 ± 0.37 a	4.83 ± 0.151 bc	9.28 ± 0.41 e	6.44 ± 0.47 a	0.245 ± 0.013 c

Different letters (a–h) in the same column indicate significant (*p* < 0.05) differences between samples for the given parameter.

**Table 4:** Nutritional labeling of vegetable creams formulated with microalgae at different levels of addition.

		STD	SP1.5	SP3	CV1.5	CV3	TC1.5	TC3	NO1.5	NO3
Energy	kcal/100 g	57.02	60.44	63.88	60.24	63.46	59.93	62.83	59.77	62.52
Fat	g/100 g	3.08	3.17	3.22	3.15	3.21	3.16	3.23	3.23	3.37
Of which saturated	g/100 g	0.36	0.39	0.42	0.39	0.41	0.44	0.52	0.51	0.66
Of which unsaturated	g/100 g	2.72	2.78	2.8	2.76	2.8	2.72	2.71	2.72	2.71
Carbohydrates	g/100 g	3.75	3.91	4.05	4.08	4.81	3.85	3.95	3.87	3.98
Of which sugars	g/100 g	1.24	1.25	1.27	1.27	1.29	1.27	1.29	1.23	1.21
Protein	g/100 g	2.5	3.04	3.59	2.72	2.95	2.92	3.34	2.81	3.13
Protein	g/100 g, N × 6.25	2.79	3.33	3.45	2.66	2.55	2.95	3.6	2.98	2.79
Salt	g/100 g	0.55	0.55	0.56	0.55	0.55	0.56	0.58	0.63	0.71
Fiber	g/100 g	2.19	2.19	2.19	2.36	2.53	2.27	2.35	2.3	2.42
Protein contribution to the energy value	% kcal	17.51% ^1^	20.12%^2^	22.46% ^2^	18.10% ^1^	18.62% ^1^	19.47% ^1^	21.26% ^2^	18.82% ^1^	20.02% ^2^
Fiber–energy ratio	g/100 kcal	3.85	3.63	3.43	3.92	3.99	3.8	3.75	3.86	3.86

^1^ “Source of protein” according to regulation (EC) No 1924/2006. ^2^ “High protein” according to regulation (EC) No 1924/2006.

## Data Availability

Not applicable.

## Supplementary Materials

The following are available online at https://www.mdpi.com/article/10.3390/foods10112550/s1, Table S1: Chemical composition of ingredients used in vegetable creams formulations.

## References

[1] GlobeNewsWire Soup Market Size to Hit USD 21 Billion by 2027; Augmenting. https://www.globenewswire.com/en/news-release/2021/05/13/2228849/0/en/Soup-Market-Size-to-Hit-USD-21-Billion-by-2027-Augmenting-Demand-for-Convenient-Healthy-Instant-Foods-to-Boost-Market-Growth-Says-Fortune-Business-Insights.html.

[2] Aschemann-Witzel J., Peschel A.O. (2019). Consumer perception of plant-based proteins: The value of source transparency for alternative protein ingredients. Food Hydrocoll..

[3] Boukid F., Rosell C.M., Rosene S., Bover-Cid S., Castellari M. (2021). Non-animal proteins as cutting-edge ingredients to reformulate animal-free foodstuffs: Present status and future perspectives. Crit. Rev. Food Sci. Nutr..

[4] Fernández-López J., Botella-Martínez C., Vera C.N.-R.d., Sayas-Barberá M.E., Viuda-Martos M., Sánchez-Zapata E., Pérez-Álvarez J.A. (2020). Vegetable Soups and Creams: Raw Materials, Processing, Health Benefits, and Innovation Trends. Plants.

[5] Farzana T., Mohajan S., Saha T., Hossain M.N., Haque M.Z. (2017). Formulation and nutritional evaluation of a healthy vegetable soup powder supplemented with soy flour, mushroom, and moringa leaf. Food Sci. Nutr..

[6] Van Gunst A., Roodenburg A.J.C., Steenhuis I.H.M. (2018). Reformulation as an Integrated Approach of Four Disciplines: A Qualitative Study with Food Companies. Foods.

[7] Federici C., Detzel P., Petracca F., Dainelli L., Fattore G. (2019). The impact of food reformulation on nutrient intakes and health, a systematic review of modelling studies. BMC Nutr..

[8] Gressier M., Sassi F., Frost G. (2020). Healthy Foods and Healthy Diets. How Government Policies Can Steer Food Reformulation. Nutrition 1992.

[9] Domínguez Díaz L., Fernández-Ruiz V., Cámara M. (2020). An international regulatory review of food health-related claims in functional food products labeling. J. Funct. Foods.

[10] Mintel High Protein Claims Appeal to Germans of All Ages|Mintel.com. https://www.mintel.com/blog/food-market-news/high-protein-claims-appeal-to-germans-of-all-ages.

[11] European Parliament and of the Council Regulation (EC) No 1924/2006 of the European Parliament and of the Council of 20 December 2006 on Nutrition and Health Claims Made on Foods. https://eur-lex.europa.eu/legal-content/en/ALL/?uri=CELEX%3A32006R1924.

[12] Nethravathy M.U., Mehar J.G., Mudliar S.N., Shekh A.Y. (2019). Recent Advances in Microalgal Bioactives for Food, Feed, and Healthcare Products: Commercial Potential, Market Space, and Sustainability. Compr. Rev. Food Sci. Food Saf..

[13] Caporgno M.P., Mathys A. (2018). Trends in Microalgae Incorporation into Innovative Food Products with Potential Health Benefits. Front. Nutr..

[14] Ak B., Avşaroğlu E., Işık O., Özyurt G., Kafkas E., Etyemez M., Uslu L. (2016). Nutritional and Physicochemical Characteristics of Bread Enriched with Microalgae Spirulina platensis. Int. J. Eng. Res. Appl..

[15] Özyurt G., Uslu L., Yuvka I., Gökdoğan S., Atci G., Ak B., Işik O. (2015). Evaluation of the Cooking Quality Characteristics of Pasta Enriched with *Spirulina Platensis*. J. Food Qual..

[16] Lucas B.F., da Rosa A.P.C., de Carvalho L.F., de Morais M.G., Santos T.D., Costa J.A.V. (2019). Snack bars enriched with Spirulina for schoolchildren nutrition. Food Sci. Technol..

[17] Boukid F., Castellari M. (2021). Food and Beverages Containing Algae and Derived Ingredients Launched in the Market from 2015 to 2019: A Front-of-Pack Labeling Perspective with a Special Focus on Spain. Foods.

[18] Nunes M.C., Fernandes I., Vasco I., Sousa I., Raymundo A. (2020). Tetraselmis chuii as a Sustainable and Healthy Ingredient to Produce Gluten-Free Bread: Impact on Structure, Colour and Bioactivity. Foods.

[19] European Parliament Council of the European Union (2015). Regulation (EU) 2015/2283 of the European Parliament and of the Council of 25 November 2015 on novel foods, amending Regulation (EU) No 1169/2011 of the European Parliament and of the Council and repealing Regulation (EC) No 258/97 of the European Parliament and of the Council and Commission Regulation (EC) No 1852/2001. Off. J. Eur. Union.

[20] Matos Â.P., Feller R., Moecke E.H.S., de Oliveira J.V., Junior A.F., Derner R.B., Sant’Anna E.S. (2016). Chemical Characterization of Six Microalgae with Potential Utility for Food Application. JAOCS J. Am. Oil Chem. Soc..

[21] Sevgili H., Sezen S., Yılayaz A., Aktaş Ö., Pak F., Aasen I.M., Reitan K.I., Sandmann M., Rohn S., Turan G. (2019). Apparent nutrient and fatty acid digestibilities of microbial raw materials for rainbow trout (Oncorhynchus mykiss) with comparison to conventional ingredients. Algal Res..

[22] Tibbetts S.M., Whitney C.G., MacPherson M.J., Bhatti S., Banskota A.H., Stefanova R., McGinn P.J. (2015). Biochemical characterization of microalgal biomass from freshwater species isolated in Alberta, Canada for animal feed applications. Algal Res..

[23] Qazi W.M., Ballance S., Uhlen A.K., Kousoulaki K., Haugen J.E., Rieder A. (2021). Protein enrichment of wheat bread with the marine green microalgae Tetraselmis chuii–Impact on dough rheology and bread quality. LWT.

[24] Lafarga T., Mayre E., Echeverria G., Viñas I., Villaró S., Acién-Fernández F.G., Castellari M., Aguiló-Aguayo I. (2019). Potential of the microalgae Nannochloropsis and Tetraselmis for being used as innovative ingredients in baked goods. LWT.

[25] Barkallah M., Dammak M., Louati I., Hentati F., Hadrich B., Mechichi T., Ayadi M.A., Fendri I., Attia H., Abdelkafi S. (2017). Effect of Spirulina platensis fortification on physicochemical, textural, antioxidant and sensory properties of yogurt during fermentation and storage. LWT-Food Sci. Technol..

[26] Matos J., Afonso C., Cardoso C., Serralheiro M.L., Bandarra N.M. (2021). Yogurt Enriched with Isochrysis galbana: An Innovative Functional Food. Foods.

[27] Batista A.P., Niccolai A., Fradinho P., Fragoso S., Bursic I., Rodolfi L., Biondi N., Tredici M.R., Sousa I., Raymundo A. (2017). Microalgae biomass as an alternative ingredient in cookies: Sensory, physical and chemical properties, antioxidant activity and in vitro digestibility. Algal Res..

[28] Durmaz Y., Kilicli M., Toker O.S., Konar N., Palabiyik I., Tamtürk F. (2020). Using spray-dried microalgae in ice cream formulation as a natural colorant: Effect on physicochemical and functional properties. Algal Res..

[29] Lafarga T., Acién-Fernández F.G., Castellari M., Villaró S., Bobo G., Aguiló-Aguayo I. (2019). Effect of microalgae incorporation on the physicochemical, nutritional, and sensorial properties of an innovative broccoli soup. LWT.

[30] Batista A.P., Nunes M.C., Raymundo A., Gouveia L., Sousa I., Cordobés F., Guerrero A., Franco J.M. (2011). Microalgae biomass interaction in biopolymer gelled systems. Food Hydrocoll..

[31] Fradique Ḿ., Batista A.P., Nunes M.C., Gouveia L., Bandarra N.M., Raymundo A. (2010). Incorporation of Chlorella vulgaris and Spirulina maxima biomass in pasta products. Part 1: Preparation and evaluation. J. Sci. Food Agric..

[32] Verdú S., Pérez A.J., Barat J.M., Grau R. (2019). Laser backscattering imaging as a control technique for fluid foods: Application to vegetable-based creams processing. J. Food Eng..

[33] Diantom A., Curti E., Carini E., Vittadini E. (2017). Effect of added ingredients on water status and physico-chemical properties of tomato sauce. Food Chem..

[34] European Parliament and of the Council Regulation (EU) No 1169/2011 of the European Parliament and of the Council of 25 October 2011 on the Provision of Food Information to Consumers. https://eur-lex.europa.eu/legal-content/EN/ALL/?uri=CELEX%3A32011R1169.

[35] Moreira J.B., Lim L.T., Zavareze E.d.R., Dias A.R.G., Costa J.A.V., Morais M.G.d. (2019). Antioxidant ultrafine fibers developed with microalga compounds using a free surface electrospinning. Food Hydrocoll..

[36] Uribe-Wandurraga Z.N., Zhang L., Noort M.W.J., Schutyser M.A.I., García-Segovia P., Martínez-Monzó J. (2020). Printability and Physicochemical Properties of Microalgae-Enriched 3D-Printed Snacks. Food Bioprocess Technol..

[37] Ruiz-Domínguez M.C., Espinosa C., Paredes A., Palma J., Jaime C., Vílchez C., Cerezal P. (2019). Determining the Potential of Haematococcus pluvialis Oleoresin as a Rich Source of Antioxidants. Molecules.

[38] Schneider N., Fortin T.J., Span R., Gerber M. (2016). Thermophysical properties of the marine microalgae Nannochloropsis salina. Fuel Process. Technol..

[39] Uribe-Wandurraga Z.N., Martínez-Sánchez I., Savall C., García-Segovia P., Martínez-Monzó J. (2021). Microalgae fortification of low-fat oil-in-water food emulsions: An evaluation of the physicochemical and rheological properties. J. Food Sci. Technol..

[40] Bleakley S., Hayes M. (2021). Functional and Bioactive Properties of Protein Extracts Generated from Spirulina platensis and Isochrysis galbana T-Iso. Appl. Sci..

[41] Batista A.P., Nunes M.C., Fradinho P., Gouveia L., Sousa I., Raymundo A., Franco J.M. (2012). Novel foods with microalgal ingredients-Effect of gel setting conditions on the linear viscoelasticity of Spirulina and Haematococcus gels. J. Food Eng..

[42] Nourmohammadi N., Soleimanian-Zad S., Shekarchizadeh H. (2020). Effect of Spirulina (*Arthrospira platensis*) microencapsulated in alginate and whey protein concentrate addition on physicochemical and organoleptic properties of functional stirred yogurt. J. Sci. Food Agric..

[43] Gouveia L., Batista A.P., Sousa I., Raymundo A., Bandarra N.M., Papadopoulos K.N. (2008). Microalgae in novel food products. Food Chemistry Research Developments.

[44] Ruocco N., Costantini S., Guariniello S., Costantini M. (2016). Polysaccharides from the Marine Environment with Pharmacological, Cosmeceutical and Nutraceutical Potential. Molecules.

